# HPV-Independent Cervical Cancer—A New Challenge of Modern Oncology

**DOI:** 10.3390/ijms262010051

**Published:** 2025-10-15

**Authors:** Ruxandra Maria Hurjui, Ion Andrei Hurjui, Tudor Andrei Buțureanu, Diana Popovici, Elena-Roxana Avădănei, Raluca Anca Balan

**Affiliations:** 1 Department of Morpho-Functional Sciences I, Grigore T. Popa University of Medicine and Pharmacy Iasi, 16 Universitatii Street, 700115 Iasi, Romania; hurjui_ruxandra-maria@d.umfiasi.ro (R.M.H.); roxanavadanei@yahoo.com (E.-R.A.); raluca.balan@umfiasi.ro (R.A.B.); 2 Department of Medicine III, Grigore T. Popa University of Medicine and Pharmacy Iasi, 700115 Iasi, Romania; hurjui_ion-andrei@d.umfiasi.ro; 3 Department Mother and Child Medicine, Grigore T. Popa University of Medicine and Pharmacy Iasi, 700115 Iasi, Romania; dianapopovici1964@yahoo.com; 4 “Elena Doamna” Clinical Hospital of Obstetrics and Gynecology, 49 Elena Doamna Street, 700398 Iasi, Romania

**Keywords:** HPV-independent cervical squamous carcinoma, HPV-associated cervical squamous carcinoma, molecular classification of cervical cancer, HPV-independent cervical adenocarcinoma, HPV-associated cervical adenocarcinoma

## Abstract

Cervical cancer is a major global health concern with serious implications for women’s health. It is most often caused by persistent infection with high-risk human papillomavirus (HPV) types. However, about 5–11% of cervical carcinoma cases are HPV-independent, entities with their own unique set of histopathological, molecular, and clinical features. The histopathological forms of HPV-independent cervical cancer include gastric-type adenocarcinoma, clear-cell, mesonephric, and endometrioid carcinoma. Unlike HPV-associated cervical cancers, which require E6 and E7 oncogenes for their expression, HPV-independent tumors exhibit specific mutations such as *TP53*, *PIK3CA*, *KRAS*, *STK11*, and *PTEN*. These mutations lead to alternative oncogenic pathways. Diagnosis of HPV-independent cervical adenocarcinoma is often delayed because of possible misclassification as endometrial adenocarcinomas, which frequently results from inadequate HPV testing. This often leads to advanced presentation stages, higher rates of lymphovascular invasion, and, in many cases, a reduced response to chemotherapy and immunotherapy—though outcomes can vary across histotypes and selected patient subgroups—due to the immune-cold tumor microenvironment. Although these morphologic and molecular characteristics describe tumors that are very difficult to manage, PI3K/mTOR and *KRAS* inhibitors may offer potential therapeutic options for selected patients. This review focuses on the pathogenic and molecular mechanisms, histopathological features, prognosis, and therapeutic difficulties of HPV-independent cervical cancers. Moreover, it provides a comprehension of contemporary issues in diagnostic methods and some new therapeutic approaches, suggesting the need for precision medicine in this aggressive type of cervical cancer. Further studies are necessary to enhance early detection, improve treatment results, and increase survival rates for patients with HPV-independent cervical cancer.

## 1. Introduction

Worldwide, cervical cancer remains one of the most common cancers affecting women. Each year, approximately 600,000 women are newly diagnosed, and about 340,000 deaths occur from this disease [1]. These statistics are found to be the worst in low- and middle-income countries, where cost-effective screening and vaccination programs are lacking [1,2]. The persistent infection with high-risk human papilloma viruses, particularly HPV-16 and HPV-18, is strongly linked to the carcinogenesis of cervical cancer and has led to the use of HPV-based screening and prophylactic vaccination as key healthcare interventions [3,4]. Despite better screening techniques, a subset of cervical cancers remains unrelated to HPV infection; these are considered HPV-independent. These subtypes of cervical cancer raise many unanswered questions regarding their etiology, pathogenesis, and required clinical interventions.

Cervical cancer that is HPV-independent is a rare variant characterized by distinctive genetic alterations, with variable frequency depending on the region. It represents roughly 5–11% of all cases of cervical cancer [5]. Its true proportion is even lower due to more advanced HPV detection techniques [6,7,8]. This variant is often reported in association with non-squamous histopathological types, particularly gastric-type adenocarcinoma (GCA), clear-cell carcinoma, and mesonephric carcinomas. All present a cervical molecular pattern and pathology different from their HPV-associated counterparts [9]. In contrast to HPV-associated cervical cancers, which are based on viral infection and influenced by E6 and E7 oncoproteins, HPV-independent tumors seem to be related to other molecular pathways. Examples include mutations in *TP53*, *PIK3CA*, *STK11*, and *KRAS* oncogenes [10,11,12].

A key issue when analyzing HPV-independent cervical cancer is distinguishing true HPV-independent cases from those that may be falsely classified. Misclassification can result from HPV testing failures, latent infections, or low-risk non-HPV subtypes undetected by routine screening [13,14,15]. Classification is further complicated by the histopathological overlap between endocervical adenocarcinoma and endometrial adenocarcinoma, which requires the use of p16, estrogen receptor (ER), progesterone receptor (PR), and carcinoembryonic antigen (CEA) immunohistochemical markers for differentiating primary cervical malignancies from metastatic or endometrial tumors [16].

Clinically, these lesions are more difficult to detect with conventional screening methods. As a result, HPV-independent cervical cancers often present at a more advanced stage than HPV-associated cases [7]. In addition, these tumors are associated with high rates of lymphovascular invasion and resistance to standard treatments, leading to poor overall survival and higher recurrence rates. Because these tumors lack viral oncogenes, their likelihood of responding to treatments targeting HPV-driven pathways, such as immune checkpoint inhibitors, is significantly reduced [17,18].

The distinct pathobiology and aggressive traits of HPV-independent cervical cancer necessitate an urgent comprehension of its molecular features. This is essential to enable the discovery of novel diagnostic biomarkers and targeted therapeutic approaches for this subgroup of tumors. This review aims to improve the understanding of HPV-independent cervical cancer by integrating the most current research data regarding its etiology, molecular mechanisms, histopathology, prognosis, clinical data, and new therapeutic modalities, thus filling the gaps needed to improve the early detection, treatment, and overall care of patients suffering from HPV-independent cervical cancer.

This review was conducted through a systematic examination of the current literature, sourcing studies from PubMed and Google Scholar. Publications from 2003 to 2025 were considered to provide a comprehensive perspective. Key terms included “HPV-independent cervical squamous carcinoma”, “HPV-associated cervical squamous carcinoma”, “molecular classification of cervical cancer”, “HPV-independent cervical adenocarcinoma”, “*TP53*”, “lncRNAs”, along with related synonyms and variations, in order to identify all studies relevant to the topic. Priority was given to original studies, molecular research, early clinical applications, and the most up-to-date clinical guidelines. In cases where multiple publications addressed similar research questions, we relied on the latest or most comprehensive study to minimize redundancy. Only peer-reviewed, full-length articles published in English were considered for inclusion. The last search was conducted on 20 September 2025. Conference abstracts, case reports, and studies with inadequate methodological information were excluded. We evaluated the studies based on their overall quality, transparency of methods, and contribution to the understanding of HPV-independent cervical cancer.

According to the WHO 2020 classification, cervical carcinomas are divided into HPV-associated and HPV-independent types. Within the IECC framework, this group is designated as “non-HPV-associated” (NHPVA), a term equivalent to the WHO nomenclature. In this review, we follow the WHO terminology, using the term HPV-independent throughout.

## 2. Pathogenesis and Molecular Mechanisms

### 2.1. Possible Reasons for HPV-Negative Status

HPV-independent cervical cancer is a diagnostic and clinical challenge. Its distinct tumor differentiation and behavior make it different from HPV-associated tumors [19]. While some cases truly do not have any association with HPV infection, others can be falsely diagnosed due to the inability to accurately detect the primary tumor or due to the misidentification of the primary tumor [20]. There is a need to better understand the reasons for HPV negativity in order to enhance diagnostic accuracy, improve patient management, and adopt more sophisticated therapeutic approaches.

**Figure 1:**
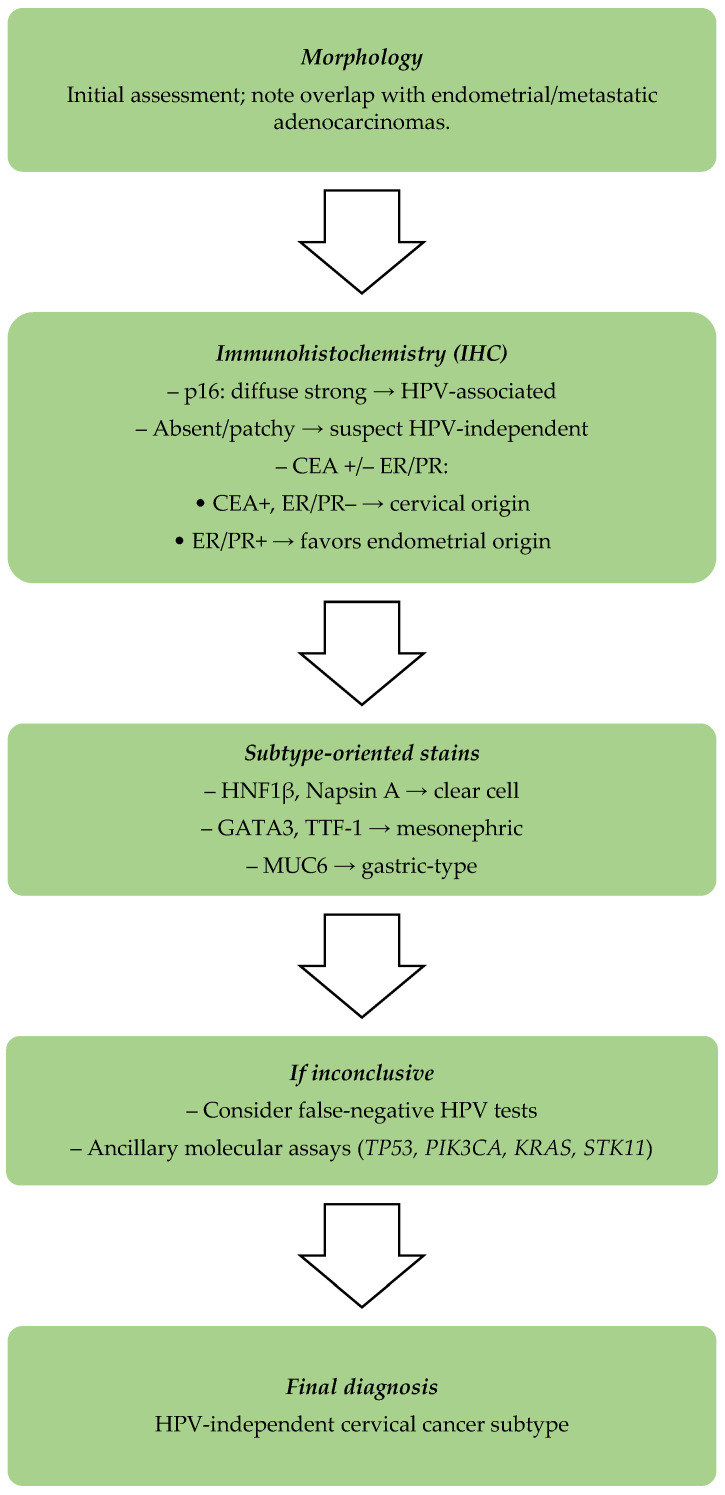
Stepwise diagnostic pathway for distinguishing HPV-independent cervical cancer from other mimics, integrating morphology, immunohistochemistry, subtype-oriented stains, and molecular assays.

A structured pathway can help reduce errors in distinguishing HPV-independent cervical cancer. Assessment begins with morphology, although overlap with endometrial or metastatic adenocarcinomas often complicates evaluation. Immunohistochemistry is the next step: strong, diffuse p16 expression supports HPV-associated tumors, while absent or patchy staining raises suspicion for HPV-independent tumors. CEA positivity in combination with p16, but the absence of ER and PR, favors a cervical origin, whereas endometrial tumors usually retain ER/PR expression and lack diffuse p16 expression. Subtype-specific stains add further clarity—HNF1β and Napsin A for clear-cell, GATA3 and TTF-1 for mesonephric, and MUC6 for gastric-type adenocarcinomas. When findings remain inconclusive, the possibility of false-negative HPV tests should be considered. Molecular testing (e.g., *TP53*, *PIK3CA*, *KRAS*, *STK11*) may provide additional diagnostic confirmation. For clarity and rapid clinical uptake, the stepwise diagnostic algorithm is also summarized in a flow diagram (Figure 1).

#### 2.1.1. Inaccurate Tumor Classification

Improper classification of tumors is one of the main reasons why some patients are diagnosed with HPV-independent cervical cancer. This issue is especially pronounced for non-cervical tumors, including those originating from metastatic lesions or the endometrium. Both endometrial and metastatic adenocarcinomas often mimic primary cervical lesions, both morphologically and clinically. Approximately 50% of cervical adenocarcinomas that do not harbor HPV have been found to be misdiagnosed cases of endometrial cancer. This indicates a need for accurate histopathological differentiation [21].

Immunohistochemical (IHC) staining is critical in the resolution of such diagnostic dilemmas. Several markers help distinguish HPV-independent cervical adenocarcinomas from endometrial adenocarcinomas [22,23].

- Cervical cancers that are HPV-independent show positive immunoexpression for CEA (carcinoembryonic antigen) and p16, but lack immunopositive results for estrogen receptor (ER) or progesterone receptor (PR) [24].
- Endometrial adenocarcinomas usually show strong ER and PR immunoexpression, but do not present diffuse p16 immunostaining [25].
- In challenging cases, additional markers such as vimentin, CD10, and MUC6 can be used for a greater diagnostic accuracy [26].
- The International Endocervical Adenocarcinoma Criteria and Classification (IECC) system has improved diagnostic precision by subclassifying adenocarcinomas into HPV-associated (HPVA) and non-HPV-associated types. The majority of HPV-independent types of cervical carcinoma are gastric-type adenocarcinomas, clear-cell carcinomas, mesonephric carcinomas, and endometrioid carcinomas, and they have distinct histological and molecular features, highlighting the importance of diagnosis based on subtypes [22,27,28].

Another possible reason for misdiagnosis could be the presence of high-grade squamous intraepithelial lesions or cervical intraepithelial neoplasia (CIN) with glandular components. These may be mistaken for adenocarcinoma in some biopsy specimens [29]. In such cases, application of p16 and Ki-67 immunostains can help identify preinvasive HPV-associated changes and clarify whether the sample is truly HPV-independent [30].

#### 2.1.2. Errors in HPV Detection Methods

HPV detection accuracy greatly influences cervical cancer classification. There are many reasons behind a false-negative HPV result, including low viral load, poor test methods, or sampling mistakes [6]. Many researchers have reported that a large number of previously negative cervical cancers were actually positive upon testing with more sensitive forms of detection, such as next-generation sequencing (NGS) [31]. The most common reasons for false-negative results are:

- Low HPV DNA copy number: Sometimes, the amount of viral load in the tumor is too low for standard PCR or hybrid capture assays to detect. This is particularly relevant when HPV has been removed from the lesion, but oncogenic changes persist [32].
- Loss of HPV DNA fragments: While the HPV genome is integrated into the host genome, the L1 region of the HPV genome, which is usually subjected to diagnostic tests, is removed. Such deletions may result in false-negative outcomes when relying on L1-based detection assays. Rates of detection may be improved by testing for E6/E7 mRNA expression, which remains active post-integration [7].
- Errors in sampling and fixation: Negative HPV results can also arise from inadequate sample collection, tissue breakdown, or flawed fixation [6].
- Variability in HPV detection methods: The sensitivities and specificities of various HPV tests impact the possibility of identifying HPV within a sample. Therefore, it is advisable to carry out multiple approaches to the detection of HPV in both clinical and investigative contexts in order to reduce false-negative cases. E6/E7 mRNA-based tests, together with NGS and enhanced sampling methods, would reduce misdiagnosis of HPV-negative cases and improve overall case accuracy [6].

#### 2.1.3. HPV Infection Latency and Non-High-Risk HPV Types

Viral latency or infection with non-high-risk HPV types could explain some cases labeled as HPV-independent cervical cancer. HPV often persists for long periods by evading immune detection and does not show typical diagnostic signs. Routine HPV tests may misidentify latent infections with low viral replication rates as false negatives [20].

Studies suggest that some low-risk HPV types may participate in cervical cancer development, even if significantly less so than the involvement of high-risk types. For example, HPV-6, HPV-11, and HPV-42, which are generally considered low-risk, have been found in some cases of cervical adenocarcinoma, suggesting that some HPV-negative cases might indeed be positive but are missed due to these viral subtypes being underrecognized [33,34]. Most HPV tests today are directed toward screening and detecting high-risk types (mostly HPV-16, 18, 31, and 33), meaning that less common genotypes likely go unidentified [33].

#### 2.1.4. Screening Challenges and Adapted Protocols

Contemporary screening frameworks prioritize detection of HPV-associated disease by using primary high-risk HPV (hrHPV) testing with cytology as triage or co-test. In the U.S., the 2024 USPSTF draft recommendation proposes cytology alone every 3 years for ages 21–29 and, for ages 30–65, primary hrHPV testing every 5 years (clinician- or patient-collected, in a clinical setting), with co-testing or cytology alone as acceptable alternatives [35]. Parallel practice updates emphasize triage biomarkers for HPV-positive results (e.g., FDA-approved p16/Ki-67 dual-stain), and 2025 ASCCP guidance formally incorporates self-collected vaginal samples for hrHPV testing obtained in clinical settings [36,37]. In global guidance, WHO continues to recommend primary HPV DNA testing as the preferred screening modality, with shorter intervals for women living with HIV (typically every 3–5 years) [38]. Emerging triage approaches, such as DNA methylation panels, are being evaluated to improve specificity after an initial positive HPV screen [39].

Because HPV-independent tumors, particularly gastric-type endocervical adenocarcinoma, often test HPV-negative and can be cytology-challenging, current HPV-centered algorithms may miss or delay detection [40,41]. Recent syntheses argue that while hrHPV-based screening reduces squamous precancer/cancer, its sensitivity for HPV-independent adenocarcinoma is limited; several groups recommend preserving cytology/endocervical sampling pathways (even when hrHPV is negative), using symptom-triggered evaluation (abnormal bleeding, watery/mucinous discharge), and considering targeted imaging (transvaginal ultrasound or MRI) for glandular lesions of uncertain origin [40,42,43]. Independently, adoption of integrated molecular diagnostics, including methylation-based triage, and, when indicated, IHC/molecular markers (e.g., MUC6 or HNF1β expression suggestive of gastric-type), in diagnostic workups could complement HPV-led programs and improve early identification of HPV-independent cases [39,44]. Maintaining these adjunctive pathways is clinically pragmatic and directly relevant to real-world diagnostic practice. Future screening guidelines should explicitly define HPV-independent pathways, including cytology-first or cytology-plus imaging in symptomatic individuals, structured referral criteria, and molecular triage strategies not reliant on viral detection. Such explicit guidance would help reduce diagnostic latency and ensure earlier recognition of this aggressive subgroup [40,41]. 

### 2.2. Mechanisms of HPV-Independent Cervical Cancer

The development of HPV-independent cervical cancer is different with regard to tumor initiation, immune interactions, molecular alterations, and clinical progression. For instance, the integration of viral oncogenes E6 and E7, which drive HPV-associated cervical cancer by inactivating tumor suppressor genes *TP53* and *RB1*, does not occur in HPV-independent cervical cancer [45].

#### 2.2.1. Immune Microenvironment Differences

The immune microenvironment plays a key role in the development and progression of HPV-associated cervical cancer. For instance, in HPV-associated cervical cancer, the presence of a viral oncoprotein triggers strong immune responses. This leads to high immune cell density in the tumor region [46]. In contrast, HPV-independent cervical cancer tends to be described as an immune-cold. This reflects lower tumor-infiltrating lymphocytes (TILs), less activation of immune checkpoints, and weaker immune surveillance [19]. HPV-independent cervical cancers generally show lower average PD-L1 expression than HPV-associated tumors, despite having more predicted neoantigens, such as nonsilent mutations, insertion–deletion events, and cancer-testis antigens. This mismatch likely makes them immune-cold. The small cohort size and diverse histologic subtypes mean that immunotherapy outcomes may not apply to all HPV-independent cases [19]. In terms of other biomarkers, the pooled prevalence of dMMR in cervical cancer is 1.9%, while high TMB (≥10 mutations/Mb) is observed in 23.7% of cases; MSI is not separately reported but generally parallels dMMR frequency [47].

Compared to other cancers, immune infiltration of helper T (CD4+) cells, cytotoxic T (CD8+) cells, and natural killer (NK) cells is often lower in HPV-independent cervical cancer. In these tumors, a marked reduction in CD8+ T cell infiltration leads to a weak and ineffective immune response that cannot eliminate tumor cells. This lack of immunogenicity may stem from the absence of viral antigens, resulting in insufficient T cell priming and activation. Additionally, lower counts of CD4+ T helper cells limit the activation of effector immune cells, causing inadequate immune response signaling and a tumor microenvironment unresponsive to immune efforts aimed at attacking the tumor [19].

**Figure 2:**
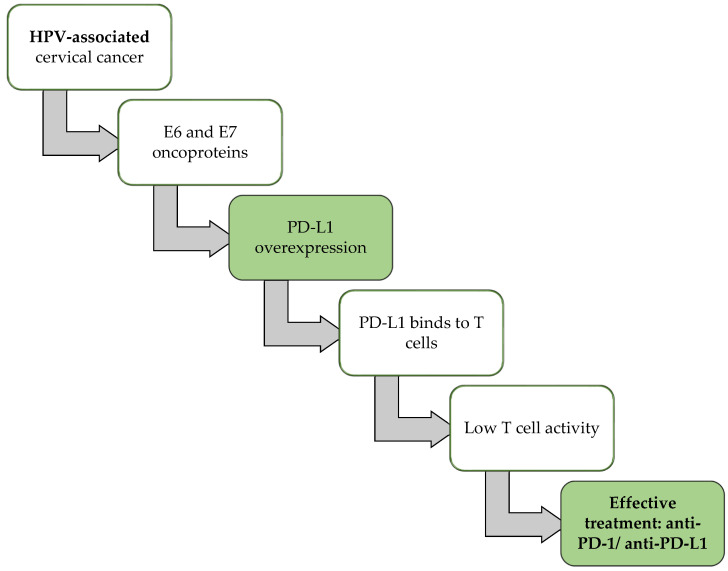
E6/E7-Mediated programmed death-ligand 1 (PD-L1) Regulation in HPV-Associated Cervical Cancer. In HPV-associated cervical cancers, the viral oncoproteins E6 and E7 induce PD-L1 overexpression, PD-L1 then binds to T cells, lowering their activity. This mechanism makes immune checkpoint inhibitors targeting programmed death-1 (PD-1) or PD-L1 particularly effective in these tumors.

An important distinction between HPV-associated and HPV-independent cancers of the uterine cervix is in the expression of programmed death-ligand 1 (PD-L1). PD-L1 is a principal immune checkpoint molecule. Its primary function is tumor immunoediting [48]. For HPV-positive patients, the oncogenic viral proteins E6 and E7 cause PD-L1 overexpression, which leads to T cell exhaustion due to the binding of programmed death-1 (PD-1) on activated T cells [49]. This makes immune checkpoint inhibitors, like anti-PD-1 and anti-PD-L1, effective in treating patients with HPV-associated cervical cancer [50]. In contrast, immune checkpoint blockade therapies tend to be less effective in HPV-independent cervical cancer, likely reflecting lower PD-L1 expression on average. The absence of viral oncogenes in HPV-independent tumors may limit sustained immune activation, and their PD-L1 microenvironment appears less dependent on chronic immune suppression. Instead, these tumors show resistance to immune activation (Figure 2) [51].

The immune evasion in HPV-independent cervical cancer can also be caused by the process of immune editing, a process in which tumor cells transform and subsequently evade the immune system’s detection through immune alteration and antigenic modulation of their tumor microenvironment [46]. In tumors that are HPV-associated, viral antigens continue to cause the system to react immunologically, which, being continuously expressed, still confers some level of recognition. In contrast, HPV-independent tumors are primarily deprived of foreign viral antigens, which subsequently facilitates the evasion of tumor immune detection through the downregulation of major histocompatibility complex class I (MHC-I) molecules, which are especially needed for severe antigen presentation to the CD8+ T lymphocytes. Due to a lack of MHC-I, cancer cells become invisible to most effector cytotoxic T-lymphocytes, thus resulting in cancer growth [52]. Moreover, the lack of costimulatory signaling molecules, such as CD80 and CD86, which are vital for the activation of T-lymphocytes, leaves most HPV-independent tumors unresponsive. The absence of these signals creates an environment where the immune system is profoundly suppressed, thereby weakening the immune system’s ability to suppress the tumor [19]. In HPV-independent tumors, downregulation of CD80/CD86 on antigen-presenting cells (APCs) reflects convergent suppressive mechanisms that blunt T-cell priming. Regulatory T cells (Tregs) remove CD80/CD86 from APC membranes through CTLA-4-dependent transendocytosis, directly depleting costimulatory ligands required for CD28 signaling [53,54]. In parallel, tumor-derived cytokines such as TGF-β (and IL-10) inhibit dendritic-cell maturation and reduce surface expression of costimulatory molecules, further limiting effective antigen presentation [55]. APC-intrinsic PD-1/PD-L1 pathways also promote a tolerogenic state; PD-L1 signaling in tumor-infiltrating dendritic cells down-modulates activation programs that include costimulatory ligand expression [56,57]. Together, these processes lead to CTLA-4-mediated ligand removal, cytokine-driven maturation arrest, and PD-L1-dependent tolerization, explaining why reduced CD80/CD86 is a recurring feature of the HPV-independent cervical cancer microenvironment and a key barrier to effective antitumor T-cell activation.

#### 2.2.2. Key Genetic Alterations

**Figure 3:**
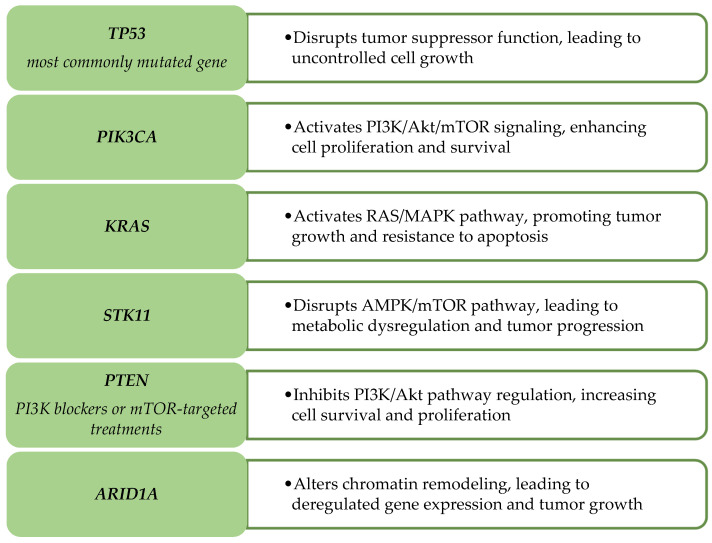
Effects on Cellular Pathways in HPV-Independent Cervical Cancer. Genetic changes in HPV-independent cervical cancer and their impact on cellular signaling pathways. These alterations drive tumor onset and progression by interfering with normal signaling networks, metabolic balance, and gene regulation.

The molecular framework of HPV-independent cervical carcinoma and HPV-associated tumors is fundamentally different, as these two types of tumors utilize distinctive drivers of cancer progression. Cervical cancer associated with HPV uses viral oncogene-mediated inactivation of the tumor suppressor genes *TP53* and *RB1* as the primary driver, while HPV-independent tumors use different patterns of somatic mutations that activate distinct oncogenic pathways leading to tumor advancement [58]. These genetic mutations largely involve the alteration of tumor suppressor genes and other mutated oncogenes, along with key signaling pathways involved in cell division, programmed cell death, and immunological evasion (Table 1). These changes may have implications for developing treatment options for patients with HPV-independent cervical cancer [59]. Figure 3 illustrates the effects on cellular pathways in HPV-independent cervical cancer.

The most commonly mutated gene in HPV-independent cervical cancer is *TP53*. This is a crucial tumor suppressor that manages the arrest of the cell cycle, apoptosis, and genomic integrity. Unlike HPV-associated tumors, where *TP53* function is inactivated by the viral E6 oncoprotein, HPV-independent tumors contain mutations within the *TP53* gene that make it nonfunctional. Such mutations lead to the expression of a nonfunctional p53 protein, which is incapable of triggering DNA repair or apoptosis in response to cellular stress, causing tumor cells to accumulate additional genetic alterations [60]. Evidence has shown missense mutations in *TP53* in a considerable fraction of HPV-independent cervical adenocarcinomas, especially in aggressive subtypes such as gastric-type adenocarcinoma and mesonephric carcinoma [61].

*PIK3CA* is another frequently mutated gene in HPV-independent cervical cancer. It encodes the catalytic subunit of phosphatidylinositol 3-kinase (PI3K), a fundamental component of the PI3K/Akt/mTOR signaling pathway. Dysregulations of the PI3K/Akt/mTOR signaling cascade are notable in the majority of cancers. *PIK3CA* mutations lead to uncontrolled cell proliferation due to the overactive PI3K pathway’s suppression of apoptosis and constitutive activation of the downstream signals [62,63,64]. Besides *TP53* and *PIK3CA* mutations, HPV-independent cervical adenocarcinomas, specifically the endometrioid and gastric-type subtypes, often exhibit mutations in the *KRAS* gene. *KRAS* is an important oncogene in the RAS/MAPK signaling pathway, regulating cellular growth and differentiation. Invasive, oncogenic mutations in *KRAS* lead to persistent signaling in the MAPK pathway, which facilitates the proliferation and sustenance of the tumor [26,65].

Alterations of *ARID1A*, previously recognized for its role in chromatin remodeling, have also been found in cases of cervical cancer that do not involve HPV infection [17]. An occult function mutation of *ARID1A* leads to epigenetic deregulation and results in overall tumor-promoting changes in gene expression [66]. Changes are also noted in the *STK11* gene, a known cervical-cancer-silenced HPV tumor suppressor. *STK11* is a multi-functional cellular sentinel that governs metabolism and energy homeostasis and serves as a ‘brake’ on tumors [67]. Changes in *STK11* are commonly found in Peutz–Jeghers syndrome, a heritable predisposition for cancer, in the absence of cervical HPV infection [68]. The loss of *STK11* function is the well-known cause for the activation of the AMPK/mTOR pathway, which, paradoxically, enables tumor cells to survive and become resistant to metabolic stress [69].

Mutations in another key tumor suppressor gene, *PTEN*, have also been observed in HPV-independent cervical carcinoma [26]. Specifically, *PTEN* acts as a negative regulator of the PI3K/Akt/mTOR pathway, such that its loss results in enhanced cell proliferation, survival, and apoptosis resistance. Notably, loss of function of *PTEN* is frequently noted in endometrioid carcinomas, which raises the possibility that HPV-independent cervical cancer and endometrial cancer may share some molecular features [70]. The existence of *PIK3CA* and *PTEN* mutations in HPV-independent tumors provides further rationale for the use of PI3K inhibitors or mTOR-directed therapeutics in some patients with these alterations [71].

The development and progression of HPV-independent cervical cancer rely profoundly on the PI3K/Akt/mTOR, WNT/β-catenin, and Hippo/YAP signaling pathways [7]. The PI3K/Akt/mTOR signaling cascade is frequently activated by *PIK3CA* alterations or by *PTEN* inactivation and is well known to promote tumor growth, angiogenesis, and metabolic reprogramming. Treatment with PI3K, AKT, or mTOR inhibitors would be valuable options in attempting to shut down this pathway [62].

The WNT/β-catenin signaling pathway has been shown to be crucial in HPV-independent cervical cancer, especially in gastric-type and mesonephric carcinomas [7]. WNT pathways are known to suffer mutations in their various parts, including *CTNNB1*, which encodes for beta-catenin, along with *APC* (Adenomatous Polyposis Coli) and *RNF43* (Ring Finger Protein 43), resulting in the uncontrolled activation of transcription associated with beta-catenin. This promotes proliferation, invasion, and resistance to treatments. WNT pathway dysregulation may explain the stem cell-like features of some cancer cells, warranting further exploration of WNT inhibitors as potential therapeutics [72].

#### 2.2.3. Participation of Long Noncoding RNAs (lncRNAs)

LncRNAs regulate gene expression, chromatin remodeling, and cellular signaling in HPV-independent cervical cancer. HPV-associated tumors exhibit viral-induced alterations in lncRNAs, whereas HPV-independent tumors rely on host lncRNAs that are tumor-suppressive and oncogenic [73].

NEF (Neighboring Enhancer of FOXA2) inhibits epithelial-to-mesenchymal transition (EMT), and its downregulation in HPV-independent tumors is linked to poor prognosis and chemotherapy resistance via TGF-β/Smad signaling [74,75]. HAND2-AS1 (Heart and Neural Crest Derivatives Expressed 2 Antisense RNA 1) suppresses angiogenesis through VEGFA signaling, with loss associated with greater vascularity and metastasis [76]. GAS5 (Growth Arrest-Specific 5) inhibits PI3K/Akt/mTOR signaling; reduced expression drives proliferation and chemoresistance in HPV-independent tumors [77].

Conversely, oncogenic lncRNAs promote tumor growth and therapy resistance. *PVT1* (Plasmacytoma Variant Translocation 1) facilitates the stabilization of the MYC oncogene, which in turn enhances the proliferation of tumor cells and makes them resistant to platinum chemotherapy [78]. *MALAT1* (Metastasis-Associated Lung Adenocarcinoma Transcript 1), widely studied in other tumor types, promotes immune evasion and EMT in cervical cancer through EZH2 binding [79]. Alterations of *XIST* (X-Inactive Specific Transcript), originally described as an X-linked gene involved in X-chromosomal inactivation, have been documented in HPV-independent cervical cancer, where it represses *PTEN* and *TP53*, resulting in enhanced resistance to therapy [79].

The balance between oncogenic and tumor-suppressive lncRNAs is therefore critical in HPV-independent cervical cancer. Emerging RNA-based therapies, small-molecule antagonists, and epigenetic remodeling represent potential strategies, while lncRNA-based biomarkers may support precision diagnostics and treatment.

## 3. Histopathological Features

Cervical cancer that occurs without an infection caused by the human papillomavirus has been described as having distinct histopathological features, one of which is a higher incidence of rare histological subtypes of adenocarcinoma compared to the conventional HPV-associated squamous cell carcinoma and adenocarcinoma [18].

HPV-independent cervical carcinomas, especially adenocarcinomas, tend to exhibit more aggressive behavior than HPV-associated cases. While the majority of squamous cell carcinomas (SCCs) are linked to HPV infection, rare HPV-independent SCCs have been reported, though this distinction does not currently affect treatment strategies. Adenocarcinomas are also categorized as either HPV-associated or HPV-independent, with the latter including gastric, clear-cell, mesonephric, and true endometrioid subtypes. Many adenocarcinomas that appear endometrioid are in fact HPV-associated tumors with reduced mucin, and true endometrioid carcinomas should only be diagnosed after excluding HPV-associated tumors and other mimics, using both morphological assessment and diffuse p16 expression. Serous carcinoma is no longer included as a primary cervical tumor due to insufficient evidence of its existence [80]. Table 2 summarizes the histopathological differences in HPV-independent cervical adenocarcinomas.

Cervical SCCs that develop independently of HPV infection tend to present with symptoms similar to HPV-related SCCs but are more commonly identified at advanced stages. These tumors often display keratinizing characteristics and a higher frequency of abnormal p53 staining, suggesting possible mutations. Other genetic changes, such as alterations in *KRAS, ARID1A,* and *PTEN*, have also been documented. Notably, there are no consistent morphological traits that can clearly differentiate HPV-independent SCCs from HPV-associated forms [80]. HPV-independent SCCs account for 5–7% of cervical SCCs. They tend to occur in older patients and are managed according to the same international guidelines as HPV-associated SCC. However, available evidence suggests lower response rates to standard chemo- and radiotherapy, and, to date, no histotype-specific trials have been performed [81,82,83,84].

**Table 2:** Histopathological Differences in HPV-Independent Cervical Adenocarcinomas. This table outlines HPV-independent cervical adenocarcinoma subtypes, detailing their characteristic histological features, key biomarkers, and clinical significance for prognosis and treatment decisions.

Subtype	Histological Features	Common Biomarkers	Clinical Implications
Gastric-Type Adenocarcinoma	Pale eosinophilic cytoplasm, clear or vacuolated cells, deep stromal invasion	MUC6, HNF1β, CDX2 (p16 negative or patchy)	Aggressive, late-stage diagnosis, high metastasis rate, poor response to therapy
Clear-Cell Carcinoma	Polygonal/hobnail cells, clear cytoplasm, central nuclei	HNF1 β, Napsin A, AMACR (p16 negative or focal)	Rare, may be linked to DES exposure, poor prognosis
Mesonephric Carcinoma	Tubular/ductal/papillary structures, eosinophilic luminal secretions	GATA3, TTF-1, CD10, AMACR (p16 negative)	Highly aggressive, high recurrence, poor prognosis
Endometrioid Carcinoma	Endometrial-like glands with villoglandular, secretory, or ciliated patterns, higher-grade tumors show solid growth, mucin commonly present	p16 negative or weak expression, ER and PR often positive, PAX8 positive	Early-stage diagnosis, better prognosis compared to other cervical adenocarcinomas, hormone receptor presence indicates possible benefit from hormonal treatment.

Abbreviations: MUC6—Mucin 6, HNF1β—Hepatocyte Nuclear Factor 1-beta, CDX2—Caudal-type Homeobox Transcription Factor, p16—Cyclin-dependent kinase inhibitor 2A, AMACR—Alpha-Methylacyl-CoA Racemase, GATA3—GATA Binding Protein 3, TTF-1—Thyroid Transcription Factor-1 (also called NKX2-1), CD10—Cluster of Differentiation 10, ER—Estrogen Receptor, PR—Progesterone Receptor, PAX8—Paired Box Gene 8, DES—Diethylstilbestrol. Data sources: Gastric-type adenocarcinoma [5,7,18,75,80,85,86,87]; Clear-cell carcinoma [5,22,27,88,89,90,91,92,93,94,95,96]; Mesonephric carcinoma [5,24,88,92,94,97,98,99,100,101]; Endometrioid carcinoma [5,79,80,102].

One of the most aggressive subtypes of HPV-independent cervical cancer is gastric-type adenocarcinoma, which features pale to eosinophilic cytoplasm, clear or vacuolated cells, and the absence of HPV-related molecular features. This subtype is usually diagnosed at later stages due to deep stromal invasion as well as lymphatic and vascular space emboli/involvement [85]. In contrast to HPV-associated adenocarcinomas, gastric-type adenocarcinomas are devoid of p16 expression or, at most, demonstrate scant focal staining, whereas HPV-associated adenocarcinomas exhibit strong diffuse p16 expression. Furthermore, they express MUC6, HNF1β, and CDX2, which are indicative of gastric differentiation [86]. Gastric-type adenocarcinoma accounts for approximately 10% of all cervical adenocarcinomas [5,75] and tends to occur in women in their early fifties (average age of 50–52 years) [87]. Notably, outcomes are generally poor, with survival significantly lower than that seen in usual-type adenocarcinomas [7]. In terms of underlying biology, genomic studies reveal frequent mutations, most commonly in *TP53*, *KRAS*, and *PIK3CA*, along with alterations in *ARID1A*, *STK11*, *CDKN2A*, and *ERBB2/HER2* amplification in a proportion of cases. Despite these differences, current management is guided by disease stage rather than histotype, yet these tumors respond less favorably to conventional chemoradiation [5].

Another histological HPV-independent subtype is clear-cell carcinoma. This tumor may be associated with intrauterine exposure to diethylstilbestrol (DES) but also occurs as an isolated finding. Morphologically, it is marked by polygonal or hobnail cells with abundant clear cytoplasm and prominent central nucleoli. Immunohistochemically, it stains positively for HNF1β, Napsin A, and AMACR, while p16 is not expressed or expressed in a patchy pattern [27,88]. Clear-cell carcinoma represents 2–7% of cervical adenocarcinomas [22]. When linked to DES exposure, it tends to arise at a much younger age [89], whereas sporadic cases present across a wider age spectrum. This histotype is notably aggressive, often escaping Pap smear detection [90], and is frequently diagnosed in advanced stages, where recurrence and distant metastases can affect up to half of patients with FIGO stage II disease [91]. Regarding molecular characteristics, genetic changes are less uniform compared with other HPV-independent tumors; however, isolated findings such as *PIK3CA* mutations, *PTEN* loss, and occasional *POLE* mutations have been described, sometimes associated with PI3K/AKT/mTOR pathway activation [92,93]. Currently, no treatment strategies are tailored specifically for this subtype, so therapy typically follows general cervical cancer guidelines [94]. Reports of response to targeted or immune-based approaches remain limited to anecdotal cases, often involving tumors with *POLE* mutations or microsatellite instability, which underscores the lack of robust trial evidence in this setting [5,95,96].

The remnants of the mesonephric duct give rise to mesonephric carcinoma, one of the least common and most aggressive HPV-independent subtypes (<1% of cervical adenocarcinomas) [97,98]. Specifically, this subtype has tubular, ductal, or papillary lesions with eosinophilic luminal secretions [88]. In terms of immunohistochemical markers, GATA3, TTF-1, CD10, and AMACR are positive for mesonephric carcinoma, while p16 and hormone receptor (ER/PR) expression is usually absent [99]. It most often develops along the lateral or posterior wall of the cervix and may not present as a well-defined mass, instead showing diverse morphologies—including infiltrative, bulky, or exophytic forms [5,98]. Clinically, around 70% of cases are diagnosed at stage IB [100], with recurrence in roughly one-third of patients and early distant spread being common [100]. Genetically, activating *KRAS* mutations are characteristic, while *NRAS* alterations are less frequent; *PIK3CA* and *PTEN* mutations are generally absent [24], though defects in *ARID1A* have been described [92,101]. For management, no histotype-specific treatment recommendations exist, and care usually follows general cervical cancer guidelines [94,101]. Anecdotal reports suggest that FGFR2-mutated tumors may respond to FGFR inhibitors; however, supporting data remain sparse [5,100].

Endometrioid carcinomas differ from other HPV-independent carcinomas by their distinct immunohistochemical and molecular features. Unlike gastric-type and mesonephric tumors, they usually show strong expression of estrogen and progesterone receptors (ER/PR) together with vimentin, characteristics consistent with an endometrial origin that may extend into the cervix. Cervical carcinomas, on the other hand, are generally negative for ER, PR, and vimentin. Endometrioid tumors may also express CD10 and CEA, markers that are typically absent in HPV-independent cervical carcinomas. From an epidemiologic standpoint, this subtype is rare, and precise data on its frequency are limited when compared with other HPV-independent forms. On a molecular level, alterations in the WNT signaling cascade, especially mutations in *CTNNB1*, are common, and disruption of the TGF-β pathway by lncRNAs has also been reported [79,102]. In clinical practice, HPV-independent endometrioid carcinomas are more often detected at advanced stages compared with HPV-related tumors, typically showing deeper local infiltration and carrying a poorer outlook. Currently, no treatment strategies are tailored specifically to this subtype; instead, therapy is guided by standard cervical cancer protocols. However, molecular profiling may offer opportunities for targeted interventions going forward [5]. Genomic profiling shows distinct HPV-dependent and independent cervical cancer patterns with key prognostic and therapeutic implications (Table 3).

**Key Points**

- Diagnostic errors can arise from HPV testing limitations, viral latency, or confusion with endometrial cancer.
- Consider HPV-independent disease when encountering gastric-type, clear-cell, mesonephric, or true endometrioid carcinomas.
- Use immunostains such as p16, ER/PR, and CEA, along with molecular profiling (*TP53*, *PIK3CA*, *KRAS*), to support accurate classification.
- These tumors are often detected at advanced stages, show limited response to standard chemoradiation, and have poorer outcomes than HPV-associated tumors.

## 4. Prognosis

Compared to HPV-associated cases, the prognosis for HPV-independent cervical cancer appears to be worse in most reports due to its late-stage diagnosis, increased likelihood of metastasis, and resistance to standard therapies. Studies have shown that these tumors are much more likely to be diagnosed at advanced FIGO stages, often because of the lack of precursor lesions that would usually be identified by HPV-based screening programs [17].

In particular, subtypes like gastric-type adenocarcinoma and mesonephric carcinoma HPV-independent tumors, which commonly invade the parametrium and peritoneal cavity, have been shown to have a higher propensity for lymphatic and distant metastases. This may contribute to higher recurrence rates and poorer overall survival outcomes. The presence of *TP53*, *KRAS*, and *PIK3CA* mutations within the tumors makes them more aggressive and resistant to chemotherapy and thus requires non-platinum chemotherapeutic alternatives. In addition, tumors that are HPV-independent are, in most cases, unresponsive to immunotherapy due to their immune-cold microenvironment, which further limits the possible options for treatment [7,17,20]. Table 4 provides a side-by-side overview of HPV-independent and HPV-associated cervical cancers, summarizing distinctions in stage at presentation, patterns of dissemination, treatment responsiveness, and survival outcomes.

## 5. Key Clinical Challenges

The most significant problem in diagnosing predominantly glandular types of HPV-independent cervical cancer is their molecular and histopathological overlap with endometrial carcinomas. This similarity is difficult to distinguish in a clinical setting [21]. Both malignancies often show overlapping histopathologic features, such as glandular differentiation, and share important molecular alterations, including *PIK3CA* and/or *PTEN* mutations; it can be quite challenging to differentiate primary HPV-independent cervical cancer from metastatic endometrial carcinoma [21].

Another notable impediment is the failure to identify a reliable molecular marker that can help detect HPV-independent cervical cancer. In comparison to HPV-associated tumors, which can be diagnosed using HPV DNA or mRNA tests, there is not a single test available for the diagnosis of HPV-independent cervical cancer. This continually impedes attempts for early detection and explains the reason for a greater proportion of patients being diagnosed at a late stage [20].

Moreover, there is an imperative need to develop individualized treatment plans that focus on the specific molecular features of HPV-independent tumors. Such treatment plans should combine targeted therapy approaches using PI3K/mTOR and *KRAS* inhibitors along with epigenetic modulators to enhance outcomes.

## 6. Management and Therapeutic Strategies

The management of HPV-independent cervical cancers is one of the most complex clinical issues due to its multifaceted molecular profile, its low responsiveness to established therapies, and the rapid advancement of the disease. In contrast to HPV-associated cervical cancer, which may benefit from prophylactic measures like HPV immunization and associated screening programs, there are no similar procedures for HPV-independent cervical cancers, and these patients are diagnosed rather late and in post-advanced stages [88]. Moreover, its molecular heterogeneity, immune-cold phenotype, and resistance to standard therapies make it imperative to formulate personalized management strategies. The primary approaches include surgery, radiation, and chemotherapy, targeting more recently emerging modalities such as immunotherapy, as well as other focused treatment modalities that appear to be more clinically beneficial. To ensure clarity, treatment should be discussed in two categories: established standards backed by clinical guidelines, and experimental approaches where data are restricted to small cohorts or early-stage studies.

### 6.1. Surgical Approaches

Surgical treatment remains an important intervention for patients with early-stage or localized HPV-independent cervical cancer, especially if the lesions are limited to the cervix. The standard approach for early-stage disease (FIGO stage IA2–IIA) is radical hysterectomy with pelvic lymphadenectomy [106]. However, a significant proportion of these women with HPV-independent cervical cancer, owing to their aggressive behavior, present at an advanced stage, thereby limiting the surgery’s curative role.

Most HPV-independent cervical cancers, like gastric-type adenocarcinoma, clear-cell carcinoma, and mesonephric carcinoma, frequently present at an advanced stage. These tumors often show deep stromal invasion, lymphovascular space involvement, and peritoneal spread at the time of diagnosis. As a result, surgical resection is usually inadequate for disease control, making adjuvant therapies necessary.

There is a higher chance of early metastasis and recurrence in HPV-independent tumors compared to their HPV-associated counterparts. Therefore, experiments with trachelectomy in fertility-sparing surgeries have been conducted for locally advanced HPV-independent cervical cancers [108]. These interventions constitute the accepted standard in early-stage cases; however, their effectiveness is frequently constrained by delayed diagnosis and the inherently aggressive course of the disease.

### 6.2. Radiotherapy and Chemotherapy

Cervical cancers that are advanced and metastatic, local or distant, are treated with wide-field external beam radiotherapy with or without brachytherapy and chemotherapy as palliative treatment [109]. Like all other matters, it is most effective when used for HPV-associated cases. However, in the case of an HPV-independent cervical cancer, the efficacy diminishes significantly.

The lack of viral oncoproteins, especially E6 and E7, in HPV-independent cervical cancers, plays a substantial role in radiotherapy resistance. HPV-independent tumors carry either wild-type or mutant p53, which enhances their ability to respond to radiation by repairing DNA damage and minimizing apoptosis. This may help explain the lower rates of locoregional control that are noted in HPV-independent cervical cancers as compared to their counterparts post radiotherapy [110].

Likewise, chemoresistance in HPV-independent cervical cancer can be challenging. As noted in the literature, studies indicate that HPV-independent tumors have lower mRNA levels for genes coding for proteins responsible for the reaction to DNA damage, resulting in a reduced ability to undergo apoptosis following chemotherapy with platinum drugs [111]. Moreover, the activation of pro-survival signaling pathways, such as PI3K/Akt/mTOR and RAS/MAPK, leads to both intrinsic and acquired forms of resistance to chemotherapy [112]. Due to these challenges, new approaches, such as combining chemotherapy with targeted therapy, are being investigated to improve patient outcomes. Chemoradiation continues to be part of standard guideline-based care, but its effectiveness in HPV-independent tumors is considerably reduced, highlighting the need for alternative approaches.

### 6.3. Targeted Therapy and Immunotherapy

At present, surgery and chemoradiation remain the only evidence-based standards for HPV-independent cervical cancer. No targeted or immunotherapeutic agents have yet reached guideline inclusion, and all such strategies should be considered experimental, with available data confined to early-phase studies or small patient cohorts.

The conventional chemotherapy and radiotherapy approaches appear to be less effective than anticipated, and therefore, much focus is put on targeted therapies and immunotherapy approaches for the treatment of HPV-independent cervical cancer. HPV-associated cervical cancer has responded favorably to immune checkpoint inhibitors like Pembrolizumab (anti-PD-1) and Nivolumab (anti-PD-1), while immune-cold phenotypes do not respond to current immunotherapies [113]. Nonetheless, novel strategies are being developed in order to modify the immune system to counteract this resistance.

One of the most extensively studied targeted approaches for HPV-independent cervical cancer is the inhibition of the PI3K/Akt/mTOR pathway. Considering the high frequency of *PIK3CA* mutations and *PTEN* loss in HPV-independent tumors, targeted therapies with Alpelisib, an inhibitor of PI3K, Capivasertib, an AKT inhibitor, as well as Everolimus and Temsirolimus, mTOR inhibitors, are being tested in clinical trials [114,115,116]. So far, these approaches are experimental only and have not received regulatory approval for use in cervical cancer.

Early prospective data in cervical cancer come primarily from the dual PI3K/mTOR inhibitor WX390 combined with the PD-1 inhibitor toripalimab. In a 2024 phase Ib/II multicenter study of advanced/metastatic cervical cancer, 18 patients were evaluable for efficacy, yielding an objective response rate (ORR) of 44.4% (8/18) and disease control rate (DCR) of 83.3% (15/18); the abstract did not report progression-free survival (PFS) or overall survival (OS) [117]. A related 2024 trial record describes the same combination with DCR as the primary endpoint, and an affiliated listing specifies enrollment of gastric-type adenocarcinoma, the prototypical HPV-independent subtype, though full outcome readouts for this subtype are still pending [118,119]. Outside of WX390, a 2024 prospective multicenter alpelisib program in PIK3CA-mutated gynecologic cancers (*n* = 36) reported ORR of 28% and DCR of 61% overall; the cohort included a small cervical subset but did not provide cervical-specific or HPV-stratified outcomes, nor PFS/OS estimates by histotype [120]. Collectively, these 2024–2025 reports constitute the core clinical signal for PI3K/mTOR targeting in cervical cancer, with ongoing studies increasingly enriching for HPV-independent biology [5]. Targeting the PI3K/mTOR pathway in HPV-independent cervical cancer should still be regarded as experimental, with biomarker-driven prospective trials needed before it can be integrated into routine practice.

The current evidence base is early-phase and underpowered. Sample sizes are small (e.g., *n* = 18 in the WX390 analysis), and study designs are single-arm without randomization. PFS/OS are often unreported at the abstract stage [117]. Cross-study comparability is limited. Selection criteria are heterogeneous, prior-therapy mixes vary, and reporting of HPV status is inconsistent. This is crucial because the biology and immunogenicity of HPV-independent tumors differ [5]. Moreover, the alpelisib series spans multiple gynecologic sites and does not separate outcomes for the cervical/HPV-independent subgroup. This limits any site-specific inference [120]. Given these constraints and nuances like *PIK3CA* mutation-site biology and class-specific toxicities (e.g., hyperglycemia with PI3Kα inhibitors), future trials should enrich for HPV-independent histotypes. They should use biomarker-based selection (e.g., *PIK3CA/PTEN* alterations) and include prespecified endpoints (ORR, DCR, median PFS/OS) with stratification by HPV status. This will help generate decision-grade evidence [5,120].

*KRAS* mutations are relatively common in gastric-type adenocarcinomas and mesonephric carcinomas, making *KRAS*-targeted therapy a subject of interest. The *KRAS* G12C inhibitor, Sotorasib, has shown benefit in patients with non-small-cell lung cancer and colon cancer. However, its role in treating HPV-independent cervical cancer is being studied [121].

Considering the low levels of PD-L1 expression alongside a decrease in T-cell infiltration in HPV-independent cervical cancer, new immune checkpoint blockade methods may be warranted. Combining immune checkpoint inhibitors (ICIs) with epigenetic modulators like Decitabine and Vorinostat—DNMT (DNA methyltransferase) and HDAC (histone deacetylase) inhibitors, respectively—has enhanced immune recognition and response in HPV-independent tumors [122].

In HPV-independent cervical cancers, especially the gastric-type, driver mutations in *KRAS*, *PIK3CA*, and *TP53* occur frequently enough to be attractive neoantigen targets. However, the frequency, clonality, and HLA context of these mutations vary by subtype, so a universal approach is not possible [5,123]. Recent immunogenomic catalogs have mapped “public” or shared neoepitopes from recurrent hotspots in these genes and predicted their HLA class I binding. This work allows for off-the-shelf T-cell receptors (TCRs) or vaccines instead of fully bespoke products [124]. Clinically, the strongest proof-of-concept comes from KRAS programs. The lymph-node-targeted amphiphile vaccine ELI-002 has generated strong mutant-KRAS specific T-cell responses with encouraging recurrence-free survival in pancreas and colorectal cohorts [125,126]. Personalized neoantigen vaccines have shown immunogenic responses against driver-derived targets, including PIK3CA, in other solid tumors [127]. Cervical-specific trials are not yet available. Still, the overlap between HPV-independent molecular profiles and public-neoantigen libraries suggests that KRAS/PIK3CA/TP53-directed immunotherapies are technically feasible, provided studies consider subtype and HLA from the start [5,124].

**Figure 4:**
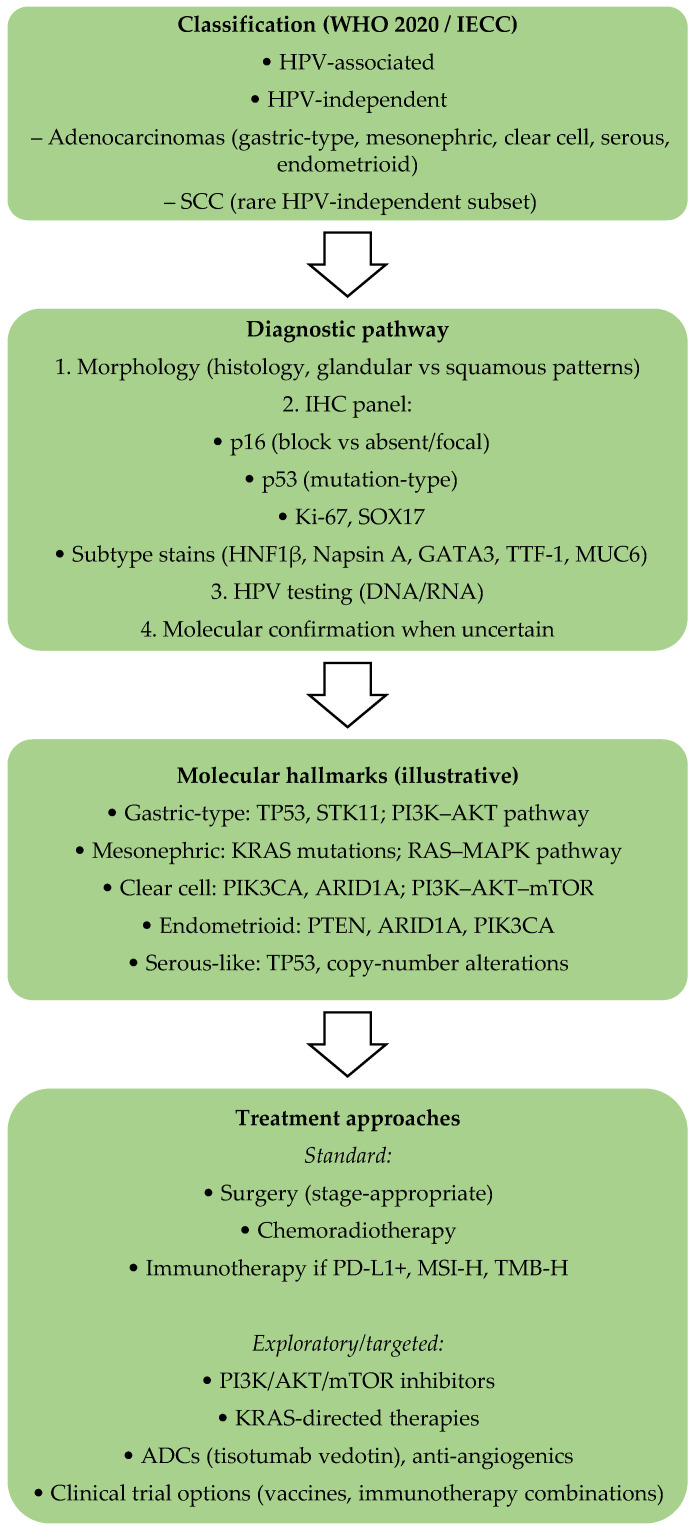
Schematic integration of WHO/IECC, diagnostic workflow, molecular hallmarks, and therapeutic approaches (standard and exploratory) in HPV-associated and HPV-independent cervical cancers.

Reviews of shared (“public”) neoantigens consistently point to *KRAS*, *TP53*, and *PIK3CA* hotspot epitopes as leading candidates for off-the-shelf vaccines and engineered TCR-T cells. They also flag practical hurdles: HLA restriction, heterogeneity between patients, and context-dependent immunodominance can blunt responses if not planned for [128,129]. For HPV-independent cervical cancer, a practical path is a hybrid approach. This approach uses a panel of shared-neoantigen vaccines (*KRAS* G12D/G12V, *PIK3CA* H1047L/E545K, *TP53* R175H/R273H) optimized for common HLA types in the target population. Where needed, it layers in personalized peptides, echoing the logic behind recent *KRAS* vaccine frameworks [125,126]. Evidence is rising that *PIK3CA* public neoantigens are immunogenic and that driver-mutation vaccines can expand durable, clonally specific T cells. These shared-epitope strategies could credibly inform a vaccine roadmap for HPV-independent cervical cancer if future trials prospectively capture HPV status, histotype, HLA typing, and immune-monitoring endpoints alongside standard clinical outcomes [128,130]. An integrated schematic is presented in Figure 4, summarizing the classification (WHO/IECC), the diagnostic pathway, the key molecular hallmarks of HPV-independent histotypes, and current versus exploratory treatment strategies.

## 7. Future Directions

Developments in molecular diagnostics, personalized therapy, and immunotherapy will likely enable advances in the management of HPV-independent cervical cancer. One of the most critical areas of research focuses on the creation of non-invasive biomarkers for tumors that are still in the preclinical stage. Some liquid biopsy techniques, such as circulating tumor DNA (ctDNA) sequencing and profiling exosomal RNAs, may be effective for earlier-stage HPV-independent tumors, thereby enhancing survival rates.

In addition to advancements in diagnostics, identifying novel therapeutic targets remains critically important for comprehensive management of HPV-independent cervical cancer. Emerging single-cell RNA-sequencing (scRNA-seq) and spatial transcriptomics stand to enormously advance understanding of the tumor microenvironment. Furthermore, there is a need for additional research into the relationship between mutation and immune microenvironment for HPV-independent tumors. Considering their immune-cold nature, strategies to turn these tumors into immunologically hot cancers such as STING (Stimulator of Interferon Genes) agonists, oncolytic viruses, and cancer vaccines—seem to be good candidate for further studies.

Antibody drug conjugates (ADCs) are reshaping systemic therapy in recurrent/metastatic cervical cancer. Following the phase III innovaTV-301 trial, tisotumab vedotin received full U.S. FDA approval as a post-chemotherapy option after demonstrating a significant overall-survival benefit versus investigator’s-choice chemotherapy [131,132,133]. Ongoing post-approval analyses and clinical updates through 2024 position tisotumab vedotin as the preferred second-line standard, with combination and earlier-line studies in progress. In patients with recurrent cervical cancer, second- or third-line therapy with tisotumab vedotin has demonstrated significantly greater efficacy compared to conventional chemotherapy [131,134]. Beyond tissue-factor targeting, HER2-directed ADCs such as trastuzumab deruxtecan (T-DXd) have shown clinically meaningful activity across gynecologic cancer, with real-world and pan-tumor data supporting benefit in HER2-expressing disease and case evidence emerging in 2025 for cervical primaries [135,136,137]. For HPV-independent histotypes, notably gastric-type adenocarcinoma, recent clinico-molecular series underscore the value of biomarker-matched approaches (HER2, PI3K/AKT/mTOR, RAS/MAPK alterations) and suggest that PD-L1 expression may have prognostic/therapeutic implications, highlighting the need for ADC- and targeted-therapy trials stratified by HPV-independent status [103,138].

Diagnostic pathways are moving beyond a binary “HPV-associated/independent” label toward integrated algorithms combining HPV DNA/RNA testing, p16 immunohistochemistry, targeted next-generation sequencing, and, where available, methylation profiling, to correctly classify HPV-independent adenocarcinomas and to surface actionable targets at diagnosis and recurrence [138,139]. Contemporary updates emphasize that p16 may be patchy or negative in HPV-independent tumors, RNA-ISH for E6/E7 can adjudicate equivocal cases, and targeted panels (with receptor assays such as HER2) refine site assignment and treatment eligibility [139]. Embedding these tools into routine workflows enables earlier trial enrollment and biomarker-guided therapy selection (e.g., HER2 for T-DXd; PI3K/AKT/mTOR or RAS/MAPK alterations for matched inhibitors), which is particularly important for the aggressive, often misclassified HPV-independent spectrum [139].

Future progress will rely on biomarker-guided clinical trials designed to test therapies specific to HPV-independent subtypes. Comprehensive multi-omic analyses, encompassing genomic, transcriptomic, and epigenetic layers, are crucial for improving classification and revealing novel therapeutic targets. Because these cancers are rare, international registries and cooperative research networks will be indispensable for pooling cases, validating biomarkers, and generating sufficiently powered evidence.

## 8. Conclusions

Cervical cancer has a distinct and difficult-to-profile subtype, HPV-independent cervical cancer, defined by molecular changes, aggressive histopathology, and resistance to conventional treatments. Unlike HPV-associated tumors, which are oncogenic because of the viral oncoprotein activity, HPV-independent tumors evade attack from the immune response through genetic mutations in oncogenes *TP53*, *PIK3CA*, *KRAS*, and other epigenetic factors. These distinctions highlight the need for developing specialized approaches for diagnosing, prognosing, and treating the disease.

The currently available treatments of choice for HPV-independent cervical cancer, such as surgery, radiotherapy, and chemotherapy, remain suboptimal and suggest a need for further development of targeted treatment options. In response, the introduction of immunologic combination therapies and those targeting PI3K/Akt/mTOR and KRAS pathways provides opportunities to improve outcomes. However, these strategies require further clinical validation and research to optimize their application and to discover biomarkers for personalized treatment approaches.

In the long run, the medical challenge due to HPV-independent cervical cancer may best be addressed through a complex strategy targeting molecular diagnostics, immunotherapy, and precision medicine. These efforts should be directed toward developing better methods for early diagnosis, increasing therapeutic efficacy, and identifying novel treatment targets with a view to improve the survival and health-related quality of life for patients suffering from this aggressive disease.

To understand the complexities described, a defining feature of HPV-independent cervical tumors is their distinct immune microenvironment, characterized by reduced infiltration of lymphocytes, diminished PD-L1 expression, and weakened antigen presentation. These characteristics underlie their poor response to standard immunotherapies and underscore the need for tailored strategies. Approaches that combine checkpoint inhibition with agents that boost immune activity, or therapies designed to restore T cell entry and functionality within the tumor microenvironment, may offer more effective solutions.

Furthermore, the recurrent genetic disruptions found in this tumor subtype—such as inactivating mutations of *TP53* and activating changes in *PIK3CA* and *KRAS*—expose therapeutic weak points that can be leveraged in treatment. Precision interventions targeting the PI3K/Akt/mTOR and RAS/MAPK cascades, modulation of WNT/β-catenin signaling, or the use of RNA-based tools against deregulated lncRNAs represent promising directions. Integrating these molecular and immunological insights into clinical practice provides a pathway toward genuinely personalized medicine for patients with HPV-independent cervical cancer.

## Figures and Tables

**Table 1:** Key Genetic Mutations in HPV-Independent Cervical Cancer. This table highlights the major genes frequently altered in HPV-independent cervical cancer (*TP53*, *PIK3CA*, *KRAS*, *STK11*, *PTEN*, and *ARID1A*), describing the types of mutations and their biological effects. These genetic changes disrupt normal cell regulation, driving tumor growth, resistance to treatment, evasion of the immune system, and loss of epigenetic control, contributing to the aggressive behavior and poor prognosis of HPV-independent cervical cancers.

Gene	Mutation Type	Relevance to Cancer Progression
*TP53*	Missense mutations	Common in HPV-independent tumors; associated with aggressive phenotypes and poor prognosis
*PIK3CA*	Activating mutations	Highly prevalent in HPV-independent tumors; a potential target for PI3K inhibitors
*KRAS*	Point mutations (G12C, G12D)	Frequent in gastric-type and mesonephric carcinomas; linked to treatment resistance
*STK11*	Loss-of-function mutations	Associated with immune evasion and poor response to therapy
*PTEN*	Loss-of-function mutations	Leads to increased tumor growth and resistance to therapy
*ARID1A*	Frameshift/Truncating mutations	Loss of function results in epigenetic deregulation, contributing to tumor aggressiveness

**Table 3:** Molecular Features, Clinical Outcomes, and Therapeutic Responses in HPV-Independent Cervical Cancer Subtypes. Values reflect 2024–2025 retrospective or small molecular datasets; absence of randomized trials and limited cohorts warrant cautious interpretation.

Subtype	Key Mutation Frequencies (Selected Genes)	Survival Outcomes	Treatment Response Rates	Sample Size/# Studies	Evidence Type/Trial Phase
Gastric-type adenocarcinoma	TP53 ~52%; TP53 73%, KRAS 46%, PIK3CA 27%, STK11 18% (small NGS subset, 2025) [103].	3-yr PFS 16.7% (HPVI) vs. 49.8% (HPVA); 3-yr OS 42.3% vs. 90.7% (definitive CCRT cohort) [104].	Definitive CCRT CR 27.8% (HPVI) vs. 81.8% (HPVA); limited targeted/IO data; HER2/PI3K-selected early reports [103,104].	NGS *n* ≈ 11; CCRT cohort *n* ≈ 40 (HPVI *n* ≈ 18); multiple small series [103,104].	Retrospective cohorts; molecular series; no randomized GAS-specific trials.
Mesonephric adenocarcinoma	KRAS 75–100% (codons 12/13); occasional PIK3CA; ARID1A/B, SMARCA4 reported [105].	5-yr OS ~74%; 5-yr PFS ~60%; high recurrence (~30%) [5,105].	Surgery ± adjuvant therapy; systemic response rates not well defined [105].	Aggregated literature ~30–60 cases; single-institution series often <20 [105].	Retrospective case series; no randomized trials.
Clear-cell carcinoma	HNF1β /Napsin A expression; ARID1A/PIK3CA/PTEN variably reported [106].	5-yr PFS ~87%; 5-yr OS ~88% (single-center cohort, *n* ≈ 49) [107].	Early-stage surgery predominant; systemic therapy/IO response not robustly quantified.	Single-center cohort *n* ≈ 49; smaller series [107].	Retrospective cohort; no randomized trials.
True endometrioid adenocarcinoma	PTEN, PIK3CA, KRAS, CTNNB1 common; TP53 variable [5].	Cervix-specific PFS/OS limited; intermediate; many NR in 2024–2025 [5].	Subtype-specific chemo/targeted/IO rates not established [5].	Sparse subtype-dedicated cohorts; embedded in mixed series [5].	Narrative/retrospective; no prospective subtype-focused trials.

Notes: CR = complete response; CCRT = concurrent chemoradiation; HPVI = HPV-independent; IO = immunotherapy.

**Table 4:** Clinical Distinctions Between HPV-Associated and HPV-Independent Cervical Cancer. The table summarizes key contrasts in stage at presentation, metastatic spread, responsiveness to chemotherapy and radiotherapy, and survival outcomes.

Feature	HPV-Associated	HPV-Independent
Stage at diagnosis	Often detected at earlier stages through HPV-based screening programs, since precursor lesions can be identified	More frequently diagnosed at advanced FIGO stages due to absence of precursor lesions and lower detectability
Nodal/peritoneal spread	Lymphovascular and nodal spread may occur but are less commonly highlighted	Higher rates of lymphovascular invasion, parametrial extension, and peritoneal or distant spread
Response to chemotherapy/radiation	Generally more responsive; viral oncogenes (E6/E7) enhance radiosensitivity and increase treatment efficacy	Reduced responsiveness; mutant/wild-type p53 aids DNA repair, while PI3K/Akt/mTOR and RAS/MAPK pathways drive resistance
Survival (stage-adjusted)	Better overall outcomes and higher survival at comparable stages	Worse prognosis with higher recurrence rates, particularly in gastric-type and mesonephric subtypes
